# Refractory Condyloma Acuminatum in Two Pediatric Patients

**DOI:** 10.7759/cureus.90123

**Published:** 2025-08-14

**Authors:** Naoki Sasaki, Haruna Yoshioka, Natsuko Saito-Sasaki, Etsuko Okada, Yu Sawada

**Affiliations:** 1 Dermatology, University of Occupational and Environmental Health, Kitakyushu, JPN; 2 Dermatology, Japan Community Healthcare Organization (JCHO) Kyushu Hospital, Kiyakyushu, JPN

**Keywords:** case report, condyloma acuminatum, human papillomavirus, recurrent, surgical resection

## Abstract

Condyloma acuminatum (CA) is thought to be a sexually transmitted disease caused by human papillomavirus, most commonly types 6 and 11. Pediatric cases are relatively rare, and the routes of transmission remain controversial. We report two cases of refractory pediatric CA involving the perianal and genital areas. Both cases were resistant to topical therapies, including imiquimod and cryotherapy, and eventually required multiple surgical interventions. Neither case presented any clinical or historical evidence suggestive of sexual abuse, and the mode of transmission remained unclear.

## Introduction

Condyloma acuminatum (CA), commonly referred to as genital warts, is an infection caused by low-risk mucosal types of human papillomavirus (HPV) [1]. Although CA is typically considered a sexually transmitted infection [2], pediatric cases can occur via non-sexual transmission routes, including perinatal transmission, horizontal transmission through household contact, and autoinoculation [3,4]. Pediatric CA is uncommon, and limited treatment options and psychosocial considerations often complicate its management. Here, we describe two cases of refractory CA in young male patients, both requiring multiple surgical procedures.

## Case presentation

Case 1

A two-year-old boy was referred to our department with persistent perianal nodules that had failed to respond to standard topical therapies. The lesions first appeared approximately three months before referral, initially as small erythematous papules on the perianal skin. Over time, they gradually increased in size and number, forming soft exophytic nodules. The patient was diagnosed with CA and was initiated on topical imiquimod 5% cream. Simultaneously, cryotherapy with liquid nitrogen was performed over three months.

Despite this intensive conservative treatment, the lesions showed no significant improvement. The lesions were frequently irritated by the use of diapers. There were no medical comorbidities, no family history of similar skin conditions, and no findings suggestive of immunodeficiency. Detailed social history, conducted through structured parental interviews and observation by both dermatologists and pediatric staff, revealed no concerns suggestive of sexual abuse or inappropriate exposure. The family dynamic was stable, and no high-risk caregivers or behavioral signs were identified.

**Figure 1:**
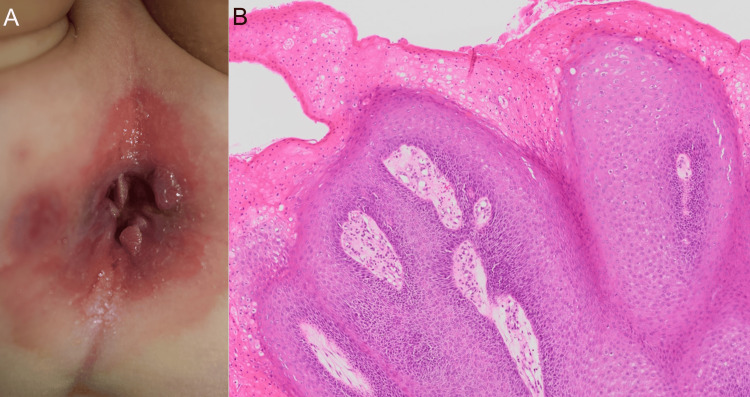
Clinical course and histopathology of case 1 (A) Initial presentation with multiple anogenital papules. (B) Histopathological examination showing papillomatosis, acanthosis, and koilocytosis in the upper epidermis, consistent with condyloma acuminatum (hematoxylin and eosin staining).

At our department, physical examination confirmed the presence of multiple nodules around the anus, without involvement of the anal canal or mucosal surfaces (Figure 1A). No abnormal anal dilation or findings indicative of chronic trauma to the anal region were observed. Serological tests for HIV, hepatitis B surface antigen (HBsAg), and anti-hepatitis C virus (anti-HCV) antibodies were performed, and all results were negative. A diagnosis of perianal CA was made. Given the lesion persistence and functional discomfort, we proposed surgical excision under sedation. After obtaining informed consent, excision with electrocautery was performed under sedation with oral chloral hydrate. The lesions were removed with minimal bleeding, and no intraoperative complications occurred.

Although the postoperative wound healed well, new papules appeared around the surgical site approximately four weeks later, indicating a limited yet definitive recurrence. A second surgical procedure was performed under the same protocol. At follow-up, no further recurrence was observed. The child remains disease-free at six months, with no visible scarring or adverse effects from anesthesia. Histopathological examination showing papillomatosis, acanthosis, and koilocytosis in the upper epidermis, consistent with CA (Figure 1B).

Case 2

A three-year-old boy presented with multiple slowly enlarging nodules involving both the perianal region and scrotum. The lesions had been first noted by the parents approximately eight months earlier and had gradually increased in both number and size. Based on the diagnosis of CA, topical imiquimod was initiated and continued for several months. However, no clinical improvement was observed. Cryotherapy with liquid nitrogen was added, and trichloroacetic acid was introduced intermittently to treat hyperkeratotic areas.

**Figure 2:**
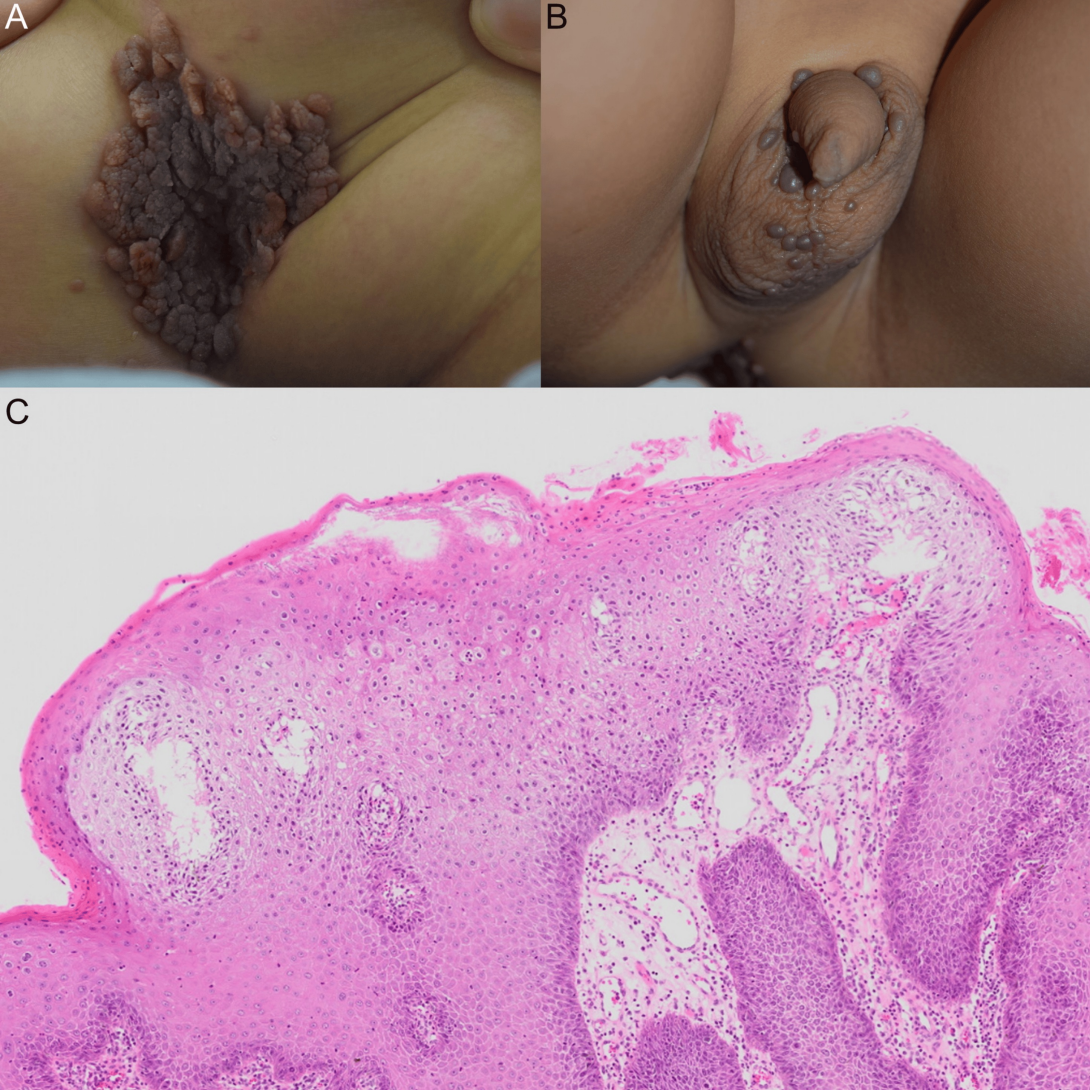
Clinical progression and histopathology of case 2 (A, B) Initial presentation of anogenital warts. (C) Histopathological examination showing acanthosis, papillomatosis, and koilocytosis of the superficial keratinocytes, consistent with condyloma acuminatum.

Despite these efforts, the lesions continued to expand, and the patient was referred to our department following a family relocation. On presentation, the patient exhibited multiple papules and nodules over the perianal skin, extending to the lateral scrotum (Figure 2A-2B). No abnormal anal dilation or findings indicative of chronic trauma to the anal region were noted on examination. Serology for HIV, HBsAg, and anti-HCV was conducted, and all results were negative. The child had a history of bronchial asthma without receiving systemic corticosteroids or immunosuppressants. Notably, topical corticosteroids had been previously applied to the perianal area for presumed diaper dermatitis.

Social and developmental assessments were conducted in collaboration with pediatric staff. No indicators of abuse or neglect were observed. The patient’s behavior, parent-child interactions, and environmental background were appropriate for his age. No similar lesions were reported among family members or caregivers, and there was no documented maternal history of genital warts.

Given the refractory course and lesion burden, surgical excision under general anesthesia was conducted. After an informed discussion with the family, the first surgical procedure was performed, and all visible lesions were excised and cauterized. Histopathology confirmed CA without dysplasia (Figure 2C).

However, recurrence was noted at the surgical margins within two months. Additional surgeries were required at 3-month intervals due to repeated recurrence, totaling four operations. Each procedure involved wide local excision and electrocautery of the lesion bases. After the fourth surgery, the lesions resolved completely, and no recurrence has been observed during the six-month follow-up period. The patient experienced no perioperative complications, and his development remains normal.

## Discussion

These cases prompt a reconsideration of how pediatric diagnoses are made, especially when medical findings intersect with moral judgment. While CA in children often raises concern for sexual abuse, such assumptions may overlook the possibility of non-abusive transmission [4-6].

In both cases, clinicians faced a diagnostic dilemma: whether to interpret anogenital warts as evidence of abuse or consider alternative explanations, such as perinatal, horizontal, or fomite-based HPV transmission [6]. Though increasingly supported in the literature, these routes remain underrecognized in clinical practice [7,8]. A reflexive assumption of abuse without definitive evidence risks unjust harm to families and erosion of trust.

Clinicians must balance their legal obligations with clinical uncertainty. Overreporting, like underreporting, can cause lasting damage, particularly when diagnostic tools such as HPV genotyping or structured psychosocial evaluation are unavailable.

Treatment is often problematic. Topical therapies may be ineffective, poorly tolerated, or not formally approved for use in children [9]. The lack of response to topical therapy in our patients may reflect multiple factors, including the relatively immature immune system in early childhood, mechanical irritation from diaper use, and the presence of hyperkeratotic or extensive lesions that limit topical penetration. The treatment options for anogenital warts in children include topical agents (e.g., imiquimod 5% cream), cryotherapy with liquid nitrogen, and surgical modalities such as excision, electrocautery, or CO₂ laser ablation, which are generally reserved for extensive or refractory lesions. Observation may also be considered in selected cases, as spontaneous resolution can occur due to immune-mediated clearance [10]. In our cases, topical imiquimod and cryotherapy were ineffective, and surgical excision with electrocautery was ultimately required.

While electro-surgical excision proved effective in our cases, it required repeated procedures under anesthesia [11], highlighting the need for safer, evidence-based options tailored to pediatric patients. Ultimately, these cases underscore the need for updated guidelines, nuanced diagnostic pathways, and medical education that reflects the full complexity of pediatric CA. Protecting children requires vigilance, along with fairness, humility, and the courage to acknowledge uncertainty.

Similar pediatric cases of refractory anogenital warts have been described in the literature, often requiring repeated surgical interventions [12]. Reported outcomes vary, underscoring the need for persistence in management and the exploration of novel therapeutic approaches.

## Conclusions

We encountered two children with stubborn anogenital warts that did not respond to topical treatments but eventually resolved after several rounds of surgical removal. In neither case was there any evidence of abuse, and how the virus was transmitted remained unclear. These experiences reminded us how treating pediatric CA involves not only medical judgment but also careful, compassionate consideration of the family’s circumstances.
